# The Methylation of the p53 Targets the Genes *MIR-203*, *MIR-129-2*, *MIR-34A* and *MIR-34B/C* in the Tumor Tissue of Diffuse Large B-Cell Lymphoma

**DOI:** 10.3390/genes13081401

**Published:** 2022-08-07

**Authors:** Elena N. Voropaeva, Tatjana I. Pospelova, Yuriy L. Orlov, Maria I. Churkina, Olga V. Berezina, Anna A. Gurazheva, Tatjana A. Ageeva, Olga B. Seregina, Vladimir N. Maksimov

**Affiliations:** 1 Research Institute of Internal and Preventive Medicine—Branch of Institute of Cytology and Genetics of Siberian Branch of the Russian Academy of Sciences, 630089 Novosibirsk, Russia; 2 Faculty of Advanced Training and Retraining of Doctors, Novosibirsk State Medical University of the Ministry of Health of the Russian Federation, 630091 Novosibirsk, Russia; 3 The Digital Health Institute, I.M. Sechenov First Moscow State Medical University of the Ministry of Health of the Russian Federation, 119991 Moscow, Russia; 4 Regional Center of High Medical Technologies, 630084 Novosibirsk, Russia

**Keywords:** microRNA, methylation, diffuse large B-cell lymphoma

## Abstract

The regulation of oncogenes by microRNA is a focus of medical research. hsa-miR-203, hsa-mir-129, hsa-miR-34a, hsa-miR-34b and hsa-miR-34c are oncosuppressive microRNAs that mediate the antitumor activity of p53. We seek to evaluate the frequencies, co-occurrence and clinical significance of the methylation of the *MIR-203*, *MIR-129-2*, *MIR-34A* and *MIR-34B/C* genes in the tumor tissue of diffuse large B-cell lymphoma (DLBCL). The methylation was assessed in 73 samples of DLBCL and in 11 samples of lymph nodes of reactive follicular hyperplasia by Methyl-Specific Polymerase Chain Reaction (MS-PCR) and Methylation-Sensitive High-Resolution-Melting (MS-HRM) methods. All four studied genes were not methylated in the tissue of reactive lymphatic nodes. The methylation frequencies of the *MIR-129-2*, *MIR-203*, *MIR-34A* and *MIR-34B/C* genes in lymphoma tissue were 67%, 66%, 27% and 62%, respectively. Co-occurrence of *MIR-203*, *MIR-129-2* and *MIR-34B/C* genes methylation, as well as the methylation of *MIR-34B/C* and *MIR-34A* pair genes were detected. The *MIR-34A* gene methylation was associated with increased International Prognostic Index (IPI) (*p* = 0.002), whereas the *MIR-34B/C* (*p* = 0.026) and *MIR-203* (*p* = 0.011) genes’ methylation was connected with Ki-67 expression level in tumor tissue at more than 45%. We found an increasing frequency of detection of *MIR-34A* gene methylation in the group of patients with the Germinal-Center B-cell like (GCB-like) subtype of DLBCL (*p* = 0.046). There was a trend towards a decrease in the remission frequency after the first line of therapy (*p* = 0.060) and deterioration in overall survival (OS) (*p* = 0.162) in patients with DLBCL with methylation of the *MIR-34A* promoter. The methylation of the *MIR-34A*, *MIR-34B/C*, *MIR-129-2* and *MIR-203* genes in DLBCL is tumor-specific and occurs in combination. The methylation of the studied genes may be a potential differential diagnostic biomarker to distinguish between lymphoma and reactive lymph nodes, while its independent predictive value has not been confirmed yet.

## 1. Introduction

MicroRNA presents a type of small (18–25 nucleotides) non-coding molecules of RNA. It is transcribed with genome DNA and undergoes further processing and export to cytoplasm [1]. MicroRNA action is a universal mechanism of the post-transcriptional regulation of gene expression. Several thousand of microRNAs are encoded in the human genome, forming an extensive regulatory network that is involved in a variety of signaling pathways and cellular processes. MicroRNA regulation disturbances are involved in the development of many diseases, including all type of neoplasms [2].

The p53 protein, encoded by the main oncosuppressor gene *TP53*, regulates the transcription of a range of microRNAs and thereby mediates its antitumor effects. In particular, it has been shown that the p53-mediated response to genotoxic cellular stress occurs due to the activation of the expression of oncosuppressor microRNAs [3,4]. This is realized by direct and indirect transcription mechanisms. Thus, p53-responsive elements have been identified in the genes encoding microRNAs, such as hsa-miR-34a, hsa-miR-34b/c, hsa-let-7, hsa-miR-16-2, hsa-miR-29b, hsa-miR-129 and has-miR-145, binding to which p53 induces their transcription [4,5,6,7]. The p53 protein participates in the regulation of other oncosuppressor microRNA expression, namely hsa-miR-16-1, hsa-miR-203 and hsa-miR-143, by accelerating the processing of these molecules [4,8]. 

It is known that microRNAs in hematopoietic cells provide the proper course of hematopoietic processes, and the alteration of microRNAs’ expression leads to various pathological conditions, including the development of hematological malignancies. MicroRNAs are responsible of the regulation of the immune system functioning, from maintaining the pool of stem cells to the maturation and functioning of T and B lymphocytes, and also play a more subtle role in making the cell resistant or sensitive to apoptosis, proliferation and differentiation [9].

The malfunction of DNA methylation is a central point of the evolution of tumors. Since a large number of genes encoding microRNAs are found in CpG-rich regions, it has been suggested that DNA methylation may play a significant role in their transcriptional disruption [1,10]. It was shown later that hypermethylation is the most important mechanism for the reduction in microRNA expression in tumors [10,11]. 

The aberrant methylation of microRNA genes associated with the underlying p53-signal pathway is assumed to be a potentially useful molecular biomarker in the diagnosis and prognosis of tumors and the development of strategies for the targeted therapy of malignancies [1,12]. 

According to the 2016 revision of the World Health Organization classification of lymphoid neoplasms, non-Hodgkin lymphomas are considered to be an extremely numerous group of neoplasms that include several dozens of variants [13]. DLBCL is the most common type of non-Hodgkin lymphoma (accounts for up to 80% of all cases of aggressive variants) [14]. 

In the course of a literature search, we selected p53-responsive miRNAs that meet the following criteria: reduced expression in lymphoma, oncosuppressive properties and interactions with miRNA targets important for lymphomagenesis pathways. The reduced expression of p53-regulated oncosupressive microRNAs in non-Hodgkin’s lymphomas has been described, namely hsa-miR-203 [15], hsa-mir-129 [16], hsa-miR-34a [17], hsa-miR-34b [17], hsa-miR-34c [17] and others [18,19,20,21] that may be induced by epigenetic mechanisms. 

Thus, the metylation of *MIR-34A*, *MIR-34B/C*, *MIR-203* and *MIR-129-2* genes in DLBCL has been studied in several previous studies [16,17,22]; however, unlike *MIR-34A* and *MIR-34B/C*, the methylation of *MIR-203* and *MIR-129-2* has been studied on mixed groups of non-Hodgkin lymphoma patients that included only few patients with DLBCL [16,22]. At the same time, non-Hodgkin lymphomas are a biologically and genetically heterogeneous group of diseases. In order to clarify the prevalence of methylation of these genes in DLBCL, validation on a larger group of samples is necessary. Moreover, in these studies, the peripheral blood of donors or normal bone marrow were used as controls, but not the benign lymph node tissue. Consequently, it was difficult to detect the *MIR-203* and *MIR-129-2* methylation in the lymphoid tissue of patients with lymphoma as tumor-specific.

It is unclear whether the methylation of p53-responsive microRNA genes is a combined phenomenon in lymphomas or it is an independent event. The simultaneous analysis of the methylation of *MIR-34A*, *MIR-34B/C*, *MIR-203* and *MIR-129-2* in a single group of DLBCL samples is currently not present. It should be noted that such microRNAs as hsa-miR-203, hsa-mir-129, hsa-miR-34a, hsa-miR-34b and hsa-miR-34c have common targets, namely C-MYC, CDK4, CDK6, MDM2, BCL2, MCL1, cyclin-D1, and others, related not only to regulators of the cell cycle at the G1/S phase transition checkpoint and programmed cell death, but also to DNA damage repair, cell migration, antitumor immune response and neoangiogenesis [14,15,22]. Thus, the simultaneous methylation of the *MIR-34A*, *MIR-34B/C*, *MIR-129-2* and *MIR-203* genes may have a potentially more negative effect and deregulate several functionally related genes involved in a single or several pathways of lymphomagenesis [22,23,24]. 

The aim of the study is to evaluate the frequency, co-occurrence and clinical significance of *MIR-203*, *MIR-129-2*, *MIR-34A* and *MIR-34B/C* genes methylation in the tumor tissue of DLBCL patients. 

## 2. Results

### 2.1. Methylation of MIR-203, MIR-129-9, MIR-34A and MIR-34B/C Genes in Lymph Node Tissues with Reactive Follicular Hyperplasia

None of the 11 DNA samples isolated from lymph nodes with reactive B-cell polyclonal proliferation contained methylation of *MIR-203*, *MIR-129-2*, *MIR-34A* and *MIR-34B/C* genes (Table 1).

### 2.2. The MIR-203, MIR-129-2, MIR-34A and MIR-34B/C Methylation in the Lymphoma Samples

**Figure 1:**
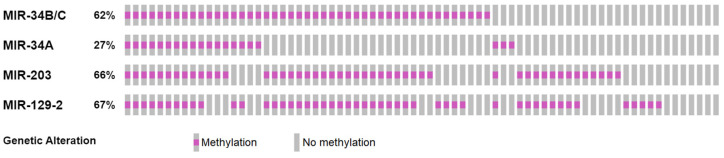
The *MIR-203*, *MIR-129-2*, *MIR-34A* and *MIR-34B/C* genes’ methylation in lymphoma samples.

The methylation of *MIR-129-2*, *MIR-203*, *MIR-34A* and *MIR-34B/C* in the DLBCL samples occurred with a frequency of 67%, 66%, 27% and 62%, respectively. As it can be seen (Figure 1), the absence of methylation of at least one of the studied genes occurred only in isolated cases. The co-occurrence of microRNA genes’ methylation was detected, exactly *MIR-203*, *MIR-129-2* and *MIR-34B/C* genes methylation, as well as the methylation of *MIR-34B/C* and *MIR-34A* pair genes (Table 2). Human Methylated and Unmethylated DNA from Control Kit (positive and negative controls) showed the expected results.

### 2.3. MIR-203, MIR-129-2, MIR-34A and MIR-34B/C Methylation Status and Clinical Features of DLBCL

The assessment of the relationship between the studied microRNA genes’ methylation and the clinical and laboratory features of the disease showed that 18/20 (90%) patients in the subgroup with *MIR-34A* gene methylation had a high and intermediate/high IPI risk (International Prognostic Index), against 26/53 (49.1%, *p* = 0.002) in the subgroup of patients without this gene methylation (Table 3). 

In the subgroup with a methylated status of this gene (*p* = 0.064), there was a tendency towards a higher frequency of detection of an increased level of LDH, which is a marker of the high paraclinical activity of the tumor. It is known that there is a correlation between this enzyme activity and the tumor volume, and the high LDH plasma level in DLBCL is a predictor of poor prognosis [25]. 

Although the mean age in these subgroups did not differ, 11/20 (55%) of DLBCL patients with *MIR-34A* methylation in tumor tissue were over 60 years old compared with 16/53 of those patients who were in the non-methylated subgroup (30.2%, *p* = 0.051).

The clinical features of DLBCL of patients with *MIR-34B/C*, *MIR-203* and *MIR-129-2* methylation did not differ from those with unmethylated status of these genes, with the exception of a higher frequency of ECOG score ≥ 2 detection in the group of patients with *MIR-34B/C* methylation.

### 2.4. Association of MIR-203, MIR-129-2, MIR-34A and MIR-34B/C Methylation with DLBCL Immunohistochemical Features

The immunohistochemical staining for Ki-67 of neoplasm tissue in lymphoma is used to assess the proliferative activity of lymphoma cells. A systematic meta-analysis including 12 studies of immunohistochemical staining for Ki-67 in DLBCL showed that the range of thresholds for the high expression of this marker, chosen by the authors, ranges from 20 to 85%, and Ki-67 overexpression is associated with high invasiveness, rapid disease progression and poor prognosis [26]. The threshold of the high expression of Ki-67 was set at the level of 45%. 

The association between *MIR-34B/C* and *MIR-203* methylation and Ki-67 expression level of more than 45% in neoplasm tissues was revealed (*p* = 0.026, OR = 3.819 (95% CI: 1.139; 12.804) and *p* = 0.011, OR = 4.457 (95% CI: 1.372; 14.481), respectively) (Table 4).

The Ki-67 expression level of more than 45% occurred in 20/26 (76.9%) in samples with a combined methylation of *MIR-34B/C* and *MIR-203*, which was significantly more frequent than in the group with the methylation of one of these genes (9/19 (47.4%, *p* = 0.041)) or in the group with the absence of *MIR-34B/C* and *MIR-203* methylation (2/9 (22.2%, *p* = 0.004)).

The immunohistochemical subtype of the tumor was determined by the Hans algorithm, which has a high concordance in results with the determination of the molecular subtype of DLBCL by gene expression profiling [27]. Out of 54 samples, 35 had non-GCB-like subtype and 19 samples had a 19 GCB-like subtype (Table 4). *MIR-34A* gene methylation was detected more often in GCB-like subtype group in comparison with the non-GCB-like subtype group (8/19 (42.1%) and 6/35 (17.1%), respectively, *p* = 0.046).

Genes *MIR-129-2* and *MIR-34A*’s methylation status was not associated with the immunohistochemical parameters of the DLBCL samples.

### 2.5. MIR-203, MIR-129-2, MIR-34A and MIR-34B/C Genes’ Methylation and Results of the Treatment of DLBCL Patients

**Figure 2:**
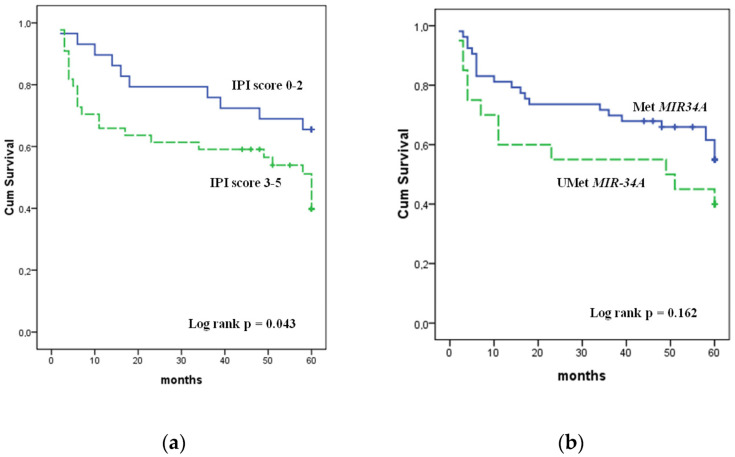
Overall survival of DLBCL patients: (**a**) IPI score 0–2 and 3–5; (**b**) with and without *MIR-34A* methylation status.

The prognostic effect of the studied genes’ methylation on the achievement of remission and overall survival rates of the studied patients was analyzed (Table 5, Figure 2).

The *MIR-34A* methylation cases demonstrated a tendency towards a decrease in the frequency of achieving remission to the first line of therapy compared with cases with unmethylated gene status—55% vs. 77.4%, respectively, *p* = 0.060. Furthermore, the IPI risk group (*p* = 0.078) was associated with the frequency of achieving remission, while the methylation status of *MIR-34B/C*, *MIR-203* and *MIR-129-2* was not associated with the effectiveness of the first-line therapy.

Only IPI had a significant impact on OS. OS was 65.5% in the low and low/intermediate IPI risk group, while it was 43.2% in the group of intermediate/high IPI risk. There was only a slight trend towards a worse survival of patients’ rate with *MIR-34A* methylation (*p* = 0.162). The Kaplan–Meier overall survival rates for patients with *MIR-34B/C*, *MIR-203* and *MIR-129-2* methylation did not differ from those with unmethylated status of these genes.

## 3. Discussion

Non-Hodgkin malignant lymphomas are a form of tumors of the hematopoietic and lymphoid tissues, which worldwide outstrips of leukemia’s in terms of the annual rate of increase in incidence. DLBCL is the most common type of non-Hodgkin malignant lymphoma. The life expectancy of patients without treatment is less than one year. The identification of additional biological markers and molecular therapeutic targets is important to improve the outcome of this disease [28].

Currently, about 200 microRNA genes that are regulated by DNA methylation have been identified in many types of neoplasia [29]. A number of microRNAs are considered to be specifically methylated in certain types of cancer. However, it is assumed that this phenomenon is partly due to a lack of knowledge of the issue and it may be clarified by studying the methylation of these genes in an extended spectrum of tumors [30].

In recent years, microRNA studies in lymphomas have revealed new opportunities for understanding the biology of the tumor process.

The methylation of DNA sites that are associated with the underlying signal p53-pathway and mediated by microRNAs is a less studied aspect in comparison with the mutational status of the *TP53* gene [31,32]. At the same time, the methylation of p53-responsive oncosuppressive microRNAs can serve as potential diagnostic and prognostic biomarker, as well as an approach to targeted treatment. 

In the current study, the frequency and clinical significance of the oncosuppressive microRNA genes’ methylation, namely *MIR-203*, *MIR-129-2*, *MIR-34A* and *MIR-34B/C* in the tumor tissue of DLBCL patients, were evaluated. 

Our results show that not only methylation of mir-34 family genes, but also the methylation of *MIR-203* and *MIR-129-2* detected in lymphoid tissues of DLBCL patients, are tumor-specific and may be potential differential diagnostic biomarkers for the distinction between lymphoma and reactive lymph nodes. 

According to our data, the present study is the first one that describes *MIR-34A*, *MIR-34B/C*, *MIR-203* and *MIR-129-2* methylation in single group of DLBCL samples. As it was shown, the absence of methylation of at least one of the studied genes occurs only in a few cases (13%). In addition, in our study, the methylation status of the analyzed genes in DLBCL tumor tissues is significantly correlated with each other, excluding pairs of *MIR-34A* and *MIR-129-2*, *MIR-34A* and *MIR-203* genes. The frequent detection of the methylation of several studied microRNA genes provides additional evidence that epigenetic instability can be an important and significant event in lymphomagenesis.

The *MIR-34A* and *MIR-34B/C* methylation frequencies in the tested DLBCL samples were 27% and 62%, respectively. This is consistent with the data of Asmar F. et al. regarding the frequency of *MIR-34A* methylation (28%) in systemic DLBCL, but, in relation to *MIR-34B/C*, it was slightly lower than the value described by the authors (78%) [17]. 

Interestingly, *MIR-34A* and *MIR-34B/C* methylation in tumor tissues of primary lymphomas of the central nervous system (PLCNS) was detected with greater frequency, namely 53/93 (57.0%) and 80/84 (95.2%) [33]. Despite the fact that most cases of PLCNS appeared to be DLBCL in histological analysis, this type of lymphoma is rightly considered as a specific biological variant. For example, it has been demonstrated that PLCNS has certain similarities in the spectrum of gene mutations with solid brain tumors, unlike other types of aggressive lymphomas [34]. This once again emphasizes the heterogeneity of lymphomas and the importance of the study of genome aberrations of each subtype.

In two earlier studies, *MIR-203* and *MIR-129-2* methylation analysis was performed on small sample sets of various hematological tumors [16,22]. It was shown that, in mixed groups of B-cell non-Hodgkin lymphoma samples, the methylation frequencies were 68.9% for *MIR-129-2* and 40.9% for *MIR-203*; however, the number of analyzed of DLBCL was quite low (2 and 13 samples) [16,22]. The obtained data clarify the frequencies of the methylation of genes encoding microRNAs miR-129 and miR-203 in DLBCL tumor tissue. They were 67% and 66%, accordingly. 

All these data indicate that the methylation of the studied genes may be one of the important mechanisms of disrupting the functioning of such p53-responsive oncosupressive microRNAs, as hsa-miR-203, hsa-mir-129, hsa-miR-34a, hsa-miR-34b and hsa-miR-34c in DLBCL. 

The limitation of our study is the relatively small number of samples that may hamper the identification of the clinical significance of *MIR-203*, *MIR-129-2*, *MIR-34A* and *MIR-34B/C* genes’ methylation in the tumor tissues of DLBCL patients. The obtained data seem to be interesting, although they do not allow us to draw unambiguous conclusions about clinical meaning of the methylation of the studied microRNA genes in lymphomas.

Our study is the first attempt to evaluate the association of *MIR-203* and *MIR-129-2* methylation with the features of DLBCL. As it was shown earlier, *MIR-129-2* and *MIR-203* methylation was not correlated with age, gender, extranodal disease or Ann Arbor staging in a mixed group of 41 patients with T-, NK- and B-cell non-Hodgkin lymphomas [16,22]. In addition, we did not observe a correlation of these two genes’ methylation with any clinical characteristics or the effectiveness of DLBCL patients’ therapy. However, there was an association between *MIR-34B/C* and *MIR-203* methylation and high level of Ki-67 expression, which is one of the widely used markers of tumor proliferation. Similar results were obtained in the research on tumors of epithelial origin. For example, another study showed a statistically significant correlation between MIR-34B/C gene methylation and Ki-67 expression in breast cancer [35]. 

There was an association of *MIR-34A* gene methylation with increased IPI (*p* = 0.002) in the studied group. In addition, we found the tendency towards an increase in the frequency of achieving remission after the first line of DLBCL therapy in patients without *MIR-34A* methylation (*p* = 0.060); at the same time, the overall survival rate did not significantly differ (*p* = 162). The *MIR-34B* methylation in studied group did not have an effect on the frequency of achieving remission and on overall survival. Thus, the obtained results are largely consistent with the previously published data.

Several previous studies have also focused on the association of miR-34 family genes’ methylation with the effectiveness of lymphoma therapy. It has shown that *MIR-34A* and *MIR34-B/C* methylated cases did not differ significantly from the *MIR-34A* and *MIR-34B/C* unmethylated DLBCL cases with respect to age, sex, clinical stage, LDH, performance score or IPI. It should be noted that, of the patients included in this study, 62 received immunochemotherapy (R-CHOP and R-CHOP-like courses with the inclusion of the targeted drug rituxamab), which is currently considered as a so-called “gold standard of therapy” of DLBCL. Cases with *MIR-34A* methylation showed a tendency towards poorer survival (*p* = 0.077) too [17]. No association between PFS or OS and *MIR-34A* or *MIR-34B/C* methylation was detected in the study of 107 PCNSL cases [34]. In these studies, it was shown that neither *MIR34A* methylation nor *TP53* mutation themselves affect survival, while the *MIR34A/TP53* “double-hit” was an independent survival factor [17,33].

DLBCLs are phenotypically and genetically heterogeneous. Previously, it was suggested to divide DLBCL by gene-expression profiling into subgroups of activated B-cell (ABC), germinal-center B-cell (GCB) and unclassified, which are associated with a differential response to targeted agents. According to our data, the differences in the expression level of the studied miRNAs in GCB and ABC molecular subtypes of DLBCL is practically not covered in the literature. However, in one recent study, miR-129-5p was significantly upregulated in the GCB subtype [36]. Gene-expression profiling is generally replaced in clinical practice by a surrogate immunohistochemical method for dividing DLBCL cases into non-GCB-like and GCB-like subgroups according to the Hans algorithm [27]. We revealed the association between *MIR-34A* methylation and the GCB-like tumor subtype in the studied samples. However, it should be noted that if we set the gene-expression profiling classification as a gold standard, the sensitivity of the Hans algorithm is described as 71% for the GCB-like and 88% for non-GCB-like subgroups [27]. Thus, the obtained data need to also be confirmed, including by the gene-expression profiling method.

## 4. Materials and Methods

### 4.1. Tumor Samples

Patients’ data and samples of tissue were collected at the Novosibirsk Regional Center of High Medical Technologies from 2015 to 2019. DNA isolation was carried out by the phenol–chloroform extraction method with guanidine from the formalin-fixed, paraffin-embedded (FFPE) tumor tissues of DLBCL patients, which contain at least 50% of malignant cells in the slice (n = 73). The DNA from FFPE biopsies of lymph nodes with reactive follicular hyperplasia (polyclonal B-cell proliferation) (n = 11) was used to assess the tumor specificity of the detected methylation.

The diagnosis of lymphoma was performed made according to the World Health Organization (WHO) Classification of lymphoid neoplasms [13]. All patients received rituximab- and anthracycline-containing combination immunochemotherapy (R-CHOP or R-CHOEP). The demographic and clinical characteristics of patients are listed in Table 3, Supplementary S1 «Clinical and methylation data». 

### 4.2. Bisulfite Conversion of DNA Samples

Total DNA was extracted from primary FFPE samples. DNA samples were treated to convert unmethylated cytosine to uracil. A total of 500 ng of DNA from each sample was subjected to bisulfite conversion using EZ DNA Methylation Kits, in accordance with the manufacturer’s protocol (Zymo research, Irvine, CA). The elution was performed with bidistilled water in a final volume of 20 μL. The Human Methylated and Unmethylated DNA Control Kit (Zymo Research, CA) was used as a control for the conversion completeness. 

### 4.3. The Assessment of Methylation Status of the MIR-34 Family Genes

**Figure 3:**
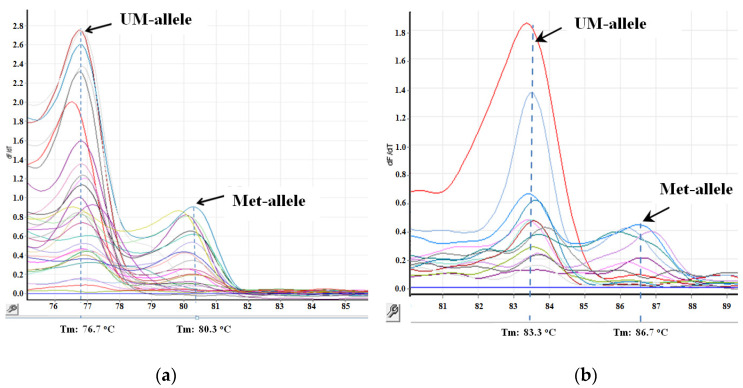
The results of the study of the methylation status of the *MIR-34B/C* (**a**) and *MIR-34A* (**b**) genes by the methyl-sensitive analysis of melting curves in clinical samples. Tm—melting point, UM-allele—unmethylated allele, Met-allele—methylated allele.

The analysis of the methylation of the *MIR-34A* and *MIR-34B/C* genes was carried out twice on a Rotor-Gene 6000 analyzer (Corbett Research, Mortlake NSW, Australia) using the MS-HRM method, in accordance with previously described methods (Figure 3) [17]. Primer sequences are listed in Table 6.

To analyze the methylation status of the *MIR-34A* gene, each reaction was performed in a final volume of 20 µL, which contained BioMaster HS-qPCR Hi-ROX SYBR (2×) (Biolabmix, Novosibirsk, Russia), 3 mM MgCl_2_, 500 nM of each primer and 10 ng of DNA modified with sodium bisulfite.

The protocol of MS-HRM analysis was as follows: one cycle 95 °C for 10 min followed by 40 repeated cycles at 95 °C for 45 s, at 64 °C for 20 s and at 72 °C for 15 s followed by one minute cycle at 65 °C and the consequent melting phase with temperature rising from 65 °C to 95 °C (temperature rise rate was 0.02 °C/s).

For *MIR-34B/C* gene analysis, each reaction was carried out in a final volume of 20 µL, which contained BioMaster HS-qPCR Hi-ROX SYBR (2×) (Biolabmix, Russia), 2 mM MgCl_2_, 300 nM of each primer and 5 ng of DNA modified with sodium bisulfite. 

The MS-HRM analysis protocol for this gene was as follows: one cycle at 95 °C for 10 min, followed by 35 repeated cycles at 95 °C for 35 s, at 56 °C for 20 s, and at 72 °C for 15 s 72 °C followed by one-minute cycle at 65 °C with the consequent melting phase with temperature rising from 65 °C to 95 °C (temperature rise rate was 0.02 °C/s). 

### 4.4. Assessment of the Methylation Status of the MIR-203 and MIR-129-2 Genes

The analysis of methylation status of the *MIR-203* and *MIR-129-2* genes was performed by MS-PCR method, in accordance with previously described methods [16,22] (Table 2).

The reaction mixture (volume 25 μL) for *MIR-203* analysis contained PCR-mix (2.5×) (Syntol, Moscow, Russia), 10 mM MgCl_2_ (volume of 3.0 μL), a mixture of deoxynucleotidetriphosphates 10 mM (volume of 0.5 μL), 1 EA SynTaq DNA-polymerase preparations (Syntol, Russia), 1 μL of each 10 mM primers and 10 ng of sodium bisulfite-modified DNA. 

The amplification protocol included the following cycles: one cycle at 95 °C for 5 min; 35 repeated cycles at 95 °C for 30 s, at 60 °C for 30 s and 72 °C for 30 s; followed by a post-elongation stage at 72 °C for 5 min.

**Figure 4:**
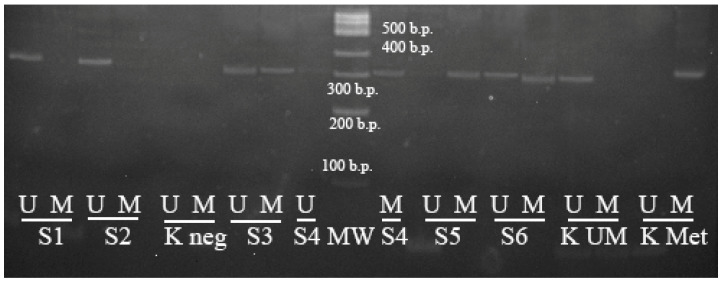
Results of the methyl-specific PCR to determine the methylation status of the *MIR-203* gene (electrophoresis in 5% polyacrylamide gel, M—PCR with primers specific for the methylated allele, UM—PCR with primers specific for the unmethylated allele). K Met—control methylated DNA, K UM—control unmethylated DNA, K neg—negative control, S1–S6—samples of DLBCL patients, MW—100 bp molecular weight marker.

To perform the electrophoretic analysis of the PCR results, 7 μL of each sample was applied on 5% polyacrylamine gel. For this purpose, a 100 b.p. molecular weight marker was used (volume of 3.0 µL). The results’ interpretation of the bands’ distribution on the electrophoregram is shown on Figure 4.

The reaction mixture (volume 25 μL) for *MIR-129-2* analysis contained PCR-mix (2.5×) (Syntol, Russia), 10 mM MgCl_2_ (volume of 3.5 μL), a mixture of deoxynucleotidetriphosphates 10 mM (volume of 0.5 μL), 1 EA SynTaq DNA-polymerase preparations (Syntol, Russia), 1.5 μL of each 10 mM primers and 8 ng of sodium bisulfite-modified DNA. 

The amplification protocol was as follows: one cycle at 95 °C for 5 min; 35 repeated cycles at 95 °C for 30 s, at temperature of primers annealing for 30 s and at 72 °C for 20 s followed by a post-elongation stage at 72 °C for 5 min. The primer annealing temperature for unmethylated sequence was 60 °C; for the methylated one, it was 62 °C and the expected length of amplicons were 188 and 189 b.p., respectively.

**Figure 5:**
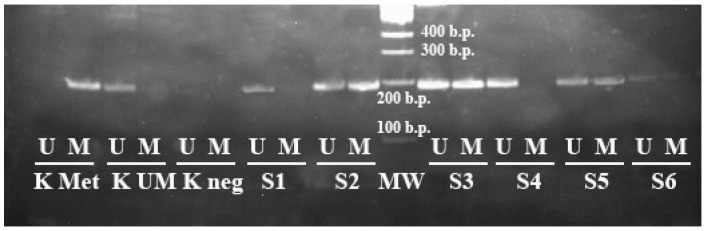
Results of the methyl-specific PCR to determine the methylation status of the *MIR-129-2* gene (electrophoresis in 5% polyacrylamide gel, M—PCR with primers specific for the methylated allele, UM—PCR with primers specific for the unmethylated allele). K Met—control methylated DNA, K UM—control unmethylated DNA, K negative—negative control, S1–S6—samples of DLBCL patients, M—100 b.p. molecular weight marker.

To perform the electrophoretic analysis of the PCR results, 7 μL of each sample was applied on 5% polyacrylamide gel. The 100 b.p. molecular weight marker was used (volume of 3.0 µL) for this purpose. The results’ interpretation of the bands’ distribution on the electrophoregram is shown on Figure 5.

### 4.5. Statistical Analysis

Statistical analysis was performed in SPSS 16.0 for Windows (IBM, New York, NY, USA). The differences were considered to be statistically significant at *p*-value < 0.05. The reliability of frequency differences genes methylation in tumor and reactive samples was evaluated by chi-squared or Fisher’s exact tests. Correlation between genes methylation status with categorical variables was estimated by a chi-squared test or Fisher’s exact test. OS was measured from the date of diagnosis to the date of last follow-up or death. Survival was plotted by the Kaplan–Meier method and compared by the log-rank test. 

The quantitative analysis of the combined methylation of the studied genes was carried out using the one-sided Fisher’s exact criterion (*p*-value) and multiple testing corrections with Benjamin–Hochberg procedure (q-value). A notion of the combined detection of the studied genes methylation was obtained using the OncoPrinter [37].

## 5. Conclusions

The high frequency of the genes’ methylation indicated that *MIR-34A*, *MIR-34B/C*, *MIR-129-2* and *MIR-203* methylation may play an important role in DLBCL development. The methylation of the *MIR-34A*, *MIR-34B/C*, *MIR-129-2* and *MIR-203* genes in DLBCL occurs in combination and is tumor-specific in lymphoma tumor tissues. The methylation of studied genes may be a potential differential diagnostic biomarker to distinguish between lymphoma and reactive lymph nodes, while its independent predictive value has not been confirmed yet. 

## Figures and Tables

**Table 1:** Methylation of the *MIR-34A*, *MIR-34B/C*, *MIR-203* and *MIR-129-2* genes’ frequencies in the tumor and control samples.

Samples	*MIR-34A*		*MIR-34B/C*		*MIR-203*		*MIR-129-2*	
	M	U	M	U	M	U	M	U
Tumor (n = 73)	20	53	45	28	48	25	49	24
Control (n = 11)	0	11	0	11	0	11	0	11
*p*-value	0.047		<0.001		<0.001		<0.001	

**Table 2:** The analysis of the tested pairs among the *MIR-34A*, *MIR-34B/C*, *MIR-203* and *MIR-129-2* genes’ methylation by the OncoPrinter.

Gene A	Gene B	*p*-Value	q-Value	Tendency
*MIR-203*	*MIR-129-2*	0.003	0.018	Co-occurrence
*MIR-34B/C*	*MIR-34A*	0.010	0.029	Co-occurrence
*MIR-34B/C*	*MIR-129-2*	0.014	0.029	Co-occurrence
*MIR-34B/C*	*MIR-203*	0.024	0.036	Co-occurrence
*MIR-34A*	*MIR-203*	0.207	0.345	Co-occurrence
*MIR-34A*	*MIR-129-2*	0.422	0.545	Mutual exclusivity

**Table 3:** Clinical parameters of the DLBCL patients of the study group.

Clinical Parameters	All Group (n = 73)	*MIR-34A*		*MIR-34B/C*		*MIR-203*		*MIR-129-2*	
		M (n = 20)	U (n = 53)	M (n = 45)	U (n = 28)	M (n = 48)	UM (n = 25)	M (n = 49)	UM (n = 24)
Sex (*p*-value)		0.100		0.698		0.101		0.562	
M	36	13	23	23	13	27	9	23	13
F	37	7	30	22	15	21	16	26	11
Age (*p*-value)		0.051		0.241		0.900		0.950	
≤60 years	46	9	37	26	20	30	16	31	15
>60 years	27	11	16	19	8	18	9	18	9
ECOG score (*p*-value)		0.758		0.034		0.617		0.457	
≤1	35	9	26	26	9	22	13	22	13
≥2	38	11	27	19	19	26	12	27	11
Stage (*p*-value)		0.513		0.068		0.095		0.105	
I	1	1	0	1	0	1	0	1	0
II	4	1	3	4	0	4	0	4	0
III	11	2	9	9	2	9	2	8	3
IV	57	16	41	31	26	34	23	36	21
Extranodal involvement (*p*-value)		0.279		0.509		0.898		0.703	
≥2 sites	14	5	9	9	5	9	5	10	4
Bone marrow involvement (*p*-value)		0.832		0.785		0.492		0.156	
Yes	17	5	12	10	7	10	7	9	8
LDH level (*p*-value)		0.064		0.305		0.202		0.270	
>450 IU/L	42	15	27	28	14	23	19	26	16
IPI score (*p*-value)		0.002		0.667		0.639		0.435	
0–2	29	2	27	17	12	20	9	21	8
3–5	44	18	26	28	16	28	16	28	16

Keys: ECOG—Eastern Cooperative Oncology Group, IPI—International Prognostic Index, LDH—Lactate dehydrogenase.

**Table 4:** Immunohistochemical parameters of the DLBCL samples.

Parameters	All Group	*MIR-34B/C* and *MIR-203*		*MIR-34A*		*MIR-34B/C*		*MIR-203*		*MIR-129-2*	
		MM	UU	M	U	M	U	M	U	M	U
IGH Ki67+ (*p*-value)	-	0.004		0.887		0.026		0.011		0.109	
>45% cells	31/54	20/26	2/9	10/17	21/37	25/37	6/17	24/34	7/20	25/39	6/15
DLBCL subtype (*p*-value)	-	-		0.046		0.541		0.851		0.851	
Non-GCB-like	35/54	-	-	6/14	29/40	21/34	14/20	23/35	12/19	23/34	12/19
GCB-like	19/54	-	-	8/14	11/40	13/34	6/20	12/35	7/19	12/34	7/19

Keys: M—methylated, U—unmethylated.

**Table 5:** The indicators of therapy effectiveness in the group of DLBCL patients.

Parameter	IPI Score			*MIR-34A*			*MIR-34B/C*			*MIR-203*			*MIR-129-2*		
	0–2	3–5	*p*-Value	M	U	*p*-Value	M	U	*p*-Value	M	U	*p*-Value	M	U	*p*-Value
Remission rate Abs (%)	24/29	28/44	0.078	11/20 (55)	41/53 (77.4)	0.060	33/45	19/28	0.616	35/48	17/25	0.660	36/49	16/24	0.547
OS (%)	65.5	43.2	0.043	40.0	56.6	0.162	53.3	50.0	0.699	54.2	48.0	0.590	57.1	41.7	0.269

Keys: M—methylated, U—unmethylated, OS—overall survival.

**Table 6:** Primer sequences and reaction conditions.

Method	Gene	Primer Sequences	Product Size (bp)	Ta (°C)	Reference
MS-HRM	*MIR-34A*	F 5/-tttttttttaggtggaggagatgt-3/ R 5/-ccaaacaaacccaaacaaaac-3/	155	64	[17]
	*MIR-34B/C*	F 5/-ttgttattaaaataaggtatagtatta-3/ R 5/-cgcttctcaaacatcttctct-3/	99	56	
MS-PCR	*MIR-203*	MF 5/-gagtattttcggtttagacgagac-3/ MR 5/-ccttttatacgacgcaaccg-3/ UMF 5/-tttgagtatttttggtttagatgagat-3/ UMR 5/-aacaccttttatacaacacaacca-3/	287	60	[22]
	*MIR-129-2*	MF 5/-gagttgggggatcgcggac-3/ MR 5/-atataccgacttcttcgattcgccg-3/	189	62	[16]
		UMF 5/-gagttgggggattgtggat-3/ UMR 5/-aatataccaacttcttcaattcacca-3/	188	60	

Keys: MS-HRM—Methylation-Sensitive High-Resolution-Melting, MS-PCR—Methylation-specific polymerase chain reaction, F—forward primer, R—reverse primer, M—methylated allele, UM—unmethylated allele, Ta—annealing temperature, bp—basic pairs.

## Data Availability

Not applicable.

## Supplementary Materials

The following are available online at https://www.mdpi.com/article/10.3390/genes13081401/s1, Supplementary S1 «Clinical and methylation data».

## Abbreviations

ABC	Activated B cell
BCL2	B-cell lymphoma-2
CDK	Cyclin-dependent kinase
DLBCL	Diffuse Large B-cell Lymphoma
ECOG	Eastern Cooperative Oncology Group
FFPE	Formalin-Fixed, Paraffin-Embedded
GCB	Germinal-Center B cell
IPI	International Prognostic Index
LDH	Lactate Dehydrogenase
MCL1	Myeloid Cell Leukemia-1
MDM2	Mouse Double Minute-2
MS-HRM	Methylation-Sensitive High-Resolution-Melting
MS-PCR	Methyl-Specific Polymerase Chain Reaction
OS	Overall Survival
PLCNS	Primary Lymphoma of the Central Nervous System
R-CHOEP	Rituximab, Cyclophosphamide, Doxorubicin, Vincristine, Etoposide, Prednisolone
R-CHOP	Rituximab, Cyclophosphamide, Doxorubicin, Vincristine, Prednisolone
WHO	World Health Organization
